# Temporal changes in pediatric tuberculosis incidence in Rio de Janeiro (2010–2023): An analysis using the Joinpoint model

**DOI:** 10.1371/journal.pone.0356811

**Published:** 2026-08-27

**Authors:** Ana Lúcia Nunes Diniz, Christine Pereira Gonçalves, Nelbe Nesi Santana Avzaradel, Saint Clair dos Santos Gomes Júnior

**Affiliations:** 1 Programa de Pesquisa Aplicada à Saúde da Criança e da Mulher, Instituto Nacional de Saúde da Mulher, da Criança e do Adolescente Fernandes Figueira, Fundação Oswaldo Cruz (Fiocruz), Rio de Janeiro, Brazil; 2 Área de Atenção Clínica à Criança e ao Adolescente, Instituto Nacional de Saúde da Mulher, da Criança e do Adolescente Fernandes Figueira, Fundação Oswaldo Cruz (Fiocruz), Rio de Janeiro, Brazil; 3 Departamento de Pesquisa e Inovação, Instituto Nacional de Saúde da Mulher, da Criança e do Adolescente Fernandes Figueira, Fundação Oswaldo Cruz (Fiocruz), Rio de Janeiro, Brazil; Para Federal University, BRAZIL

## Abstract

**Objective:**

To analyze the time trend of reported tuberculosis cases in the pediatric population in Rio de Janeiro State from 2010 to 2023.

**Methods:**

Data on new tuberculosis cases were obtained from the Notifiable Diseases Information System. Joinpoint regression analysis was used to identify inflection points and segment the time series into periods with distinct incidence trends. These segments were then used to assess statistically significant differences in the distribution of sociodemographic, clinical, and outcome variables.

**Results:**

The findings reveal significant fluctuations throughout the period, with a marked increase from 2020 to 2023, particularly among children under five years of age. During this period, a decrease in the Municipal Human Development Index and an increase in both infant mortality and the Gini index were also observed.

**Conclusion:**

Despite a decline in the number of reported cases, There was an upward trend in tuberculosis incidence among children and adolescents between 2020 to 2023 in Rio de Janeiro State, coinciding with a decline in development indicators. Although definitive causal relationships cannot be established, the observed patterns reinforce the need for robust epidemiological surveillance tailored to age-specific characteristics, as well as targeted interventions.

## Introduction

The occurrence of tuberculosis among children and adolescents has historically received limited attention. Despite being particularly vulnerable to both infection and disease, the volume of research and published studies focusing on pediatric tuberculosis remains relatively low compared to the adult population [1,2]. In Brazil, in 2020, according to the Sistema de Informação de Agravos de Notificação (SINAN, Brazilian Case Registry Database), 7.3% of new reported cases occurred in children under 19 years of age. However, the actual incidence may be higher due to underreporting, largely attributed to the challenges in diagnosing tuberculosis in this age group. This is especially true for children under 10 years old, who are typically paucibacillary and often unable to produce sputum, making it difficult to conduct standard diagnostic tests such as sputum analysis [3].

Recent studies have explored temporal trends in TB incidence, highlighting variations across population groups and geographic regions, in Brazil. Nationally, pediatric TB exhibits a heterogeneous spatial distribution and fluctuating temporal patterns, with incidence rates increasing in 2018 and 2019, followed by a sharp decline in 2020, likely due to disruptions in surveillance and diagnostic services caused by the COVID-19 pandemic [4]. An analysis of TB mortality among individuals aged 0–19 years from 1996 to 2020 revealed an average annual decrease of 2.8%, although a non-significant increase was observed between 2017 and 2020, underscoring the need for continued monitoring in this age group [5]. Regional studies, such as those conducted in the state of São Paulo, have identified high-risk areas for TB transmission, emphasizing the role of socioeconomic disparities and healthcare access in shaping incidence patterns [6].

Studies have consistently shown that social determinants such as human development indicators, access to health services, and the efficiency of health surveillance systems play a critical role in both the incidence and treatment outcomes of tuberculosis. Regions with low socioeconomic indicators tend to be more vulnerable to the disease, since poverty, poor nutrition, population density, and precarious housing conditions create an environment conducive to tuberculosis transmission. Additionally, the difficulty in accessing health services, especially in remote or peripheral areas, compromises early diagnosis and adherence to treatment, which contributes to the maintenance of the transmission chain. The weakness of epidemiological surveillance, in turn, makes it difficult to identify contacts and adequately monitor cases, negatively impacting disease control indicators. Addressing TB effectively therefore requires an intersectoral approach, that integrates targeted health interventions which broader public policies aimed at improving living conditions and reducing social inequalities. [7–10].

The period between 2010 and 2023 in Brazil was marked by significant political, economic, and public health transitions that likely influenced TB surveillance and control. Changes in federal and state health policies, economic instability, and the COVID-19 pandemic disrupted healthcare services and may have contributed to fluctuations in TB incidence. Although most time-series analyses have focused on adult populations, studies have identified trend breaks and regional disparities linked to social vulnerability, healthcare access, and surveillance capacity [11]. These broader contextual factors underscore the importance of examining pediatric TB trends within this timeframe, particularly in regions such as Rio de Janeiro State, where socioeconomic inequalities and health system challenges persist.

Given the epidemiological significance of tuberculosis and the limited analysis of pediatric data in Brazil, this study aims to examine the historical series of reported tuberculosis cases among individuals under 19 years of age in Rio de Janeiro State, Brazil, from 2010 to 2023. The study also seeks to identify potential changes in the sociodemographic, clinical, and outcome-related characteristics of these cases over time in order to better understand temporal trends and inform targeted public health strategies.

## Method

### Design, population and location

This observational study analyzed historical data on new tuberculosis cases among children and adolescents aged 0–19 years reported to the Brazilian Notifiable Diseases Information System (SINAN) between 2010 and 2023 in Rio de Janeiro State, Brazil. The data were obtained through the public online platform maintained by the Department of Informatics of the Unified Health System (DATASUS), which provides access to tabulated reports generated from the available databases. The database is available at: http://tabnet.datasus.gov.br/cgi/tabcgi.exe?sinannet/cnv/tubercrj.def (accessed May 8, 2024).

### Eligibility criteria

All notification records of new cases available in SINAN until May 8, 2024 were selected, defined based on the following criteria: confirmed cases notified in Rio de Janeiro State in the period from 2010 to 2023 in the age group of 0–19 years. The criteria for confirming the diagnosis of tuberculosis cases follow the standards of the National Tuberculosis Control Program, which include clinical, epidemiological, and bacteriological diagnoses.

### Variables

The tables generated were reorganized to specify the number of new cases for each of the years considered, stratified by: clinical form (pulmonary, extrapulmonary, pulmonary and extrapulmonary), HIV testing (yes/no), directly observed treatment (yes, no and ignored or blank), outcome (cure, lost to follow-up, death from tuberculosis, death from other causes, transfer, resistance to rifampicin, change of regimen, failure and other), sex (male/female), age group (0–4, 5–9, 10–14 and 15–19), race/skin color (white, black, mixed, yellow, indigenous and unknown or not filled in) and beneficiary of a cash transfer program (yes/no), as detailed in S1 File. The criteria for considering failure in the outcome are the persistence of positive sputum smear microscopy at the end of treatment or patients who at the beginning of treatment had a strongly positive smear microscopy and maintained this situation until the 4th month or initial positive smear microscopy followed by negative results and new positive results for 2 consecutive months from the 4th month of treatment.

Socioeconomic data on the Municipal Human Development Index (MHDI), per capita income, infant mortality rate, and Gini index in Rio de Janeiro State, covering the period from 2010 to 2023, were obtained from the Atlas of Human Development in Brazil. These data are publicly available at the electronic address http://www.atlasbrasil.org.br (accessed May 6, 2024). Because official annual MHDI values were unavailable for 2011, 2022, and 2023, these missing values were replaced using mean imputation based on the average of all available annual MHDI values. This approach allowed the inclusion of the MHDI throughout the study period and ensured consistency in the temporal analyses.

The historical series of new tuberculosis cases was analyzed using annual incidence rates per 100,000 population. Incidence rates were calculated by dividing the annual number of new tuberculosis cases among children and adolescents by the corresponding resident population in the same age group and year in Rio de Janeiro State. Population denominators were obtained from the 2024 edition of the Population Projections of Brazil and Federative Units by Sex and Age (2000–2070), produced by the Brazilian Institute of Geography and Statistics (IBGE), ensuring methodological consistency in the calculation of incidence rates throughout the study period (2010–2023). The data are publicly available at: https://www.ibge.gov.br/en/statistics/social/population.html (accessed May 8, 2024).

### Data analysis

The historical series was analyzed using the Joinpoint regression model, which identifies statistically significant changes in temporal trends by fitting segmented regression models. Prior to modeling, the incidence rates were log-transformed and modeled assuming a Poisson variance structure to stabilize variance and appropriately account for the count nature of the data. The number of joinpoints was constrained to range from 0 to a maximum of 2, with a minimum of three observations per segment to ensure robust regression estimates and avoid model overfitting. The optimal model was selected using the Monte Carlo Permutation Test with 4,499 permutations. Annual Percent Change (APC) and corresponding 95% confidence intervals (95% CI) were estimated for each trend segment. According to the APC estimates, trends were classified as increasing, decreasing, or stationary, based on their direction and statistical significance. Additionally, the Average Annual Percent Change (AAPC) was calculated for the entire study period (2010–2023) to provide a summary measure of the overall temporal trend. Statistical significance was set at *p* < 0.05. Analyses were performed using the Joinpoint Regression Program (version 5.3.0.0; National Cancer Institute, Bethesda, MD, USA).

The time segments identified by the model were used to stratify the series and compare the occurrence of significant differences between the variables sex, race/skin color, beneficiary of a cash transfer program, clinical form, HIV testing, directly observed treatment, outcome, MHDI, per capita income, infant mortality, and Gini index. Categorical variables were described as absolute and relative frequencies, while numerical variables were presented as mean and standard deviation. For the comparison between the periods, the chi-square test was used for categorical variables and one-way analysis of variance (ANOVA) was used for numerical variables. A significance level of 5% was adopted. The analysis was performed using the statistical software SPSS (IBM SPSS Statistics para Windows, versão 23.0. Armonk, NY: IBM Corp.).

### Ethical issues

As this was a study that used data in the public domain and without the identification of the participants, it was not necessary to submit it to a Research Ethics Committee.

## Results

**Fig 1:**
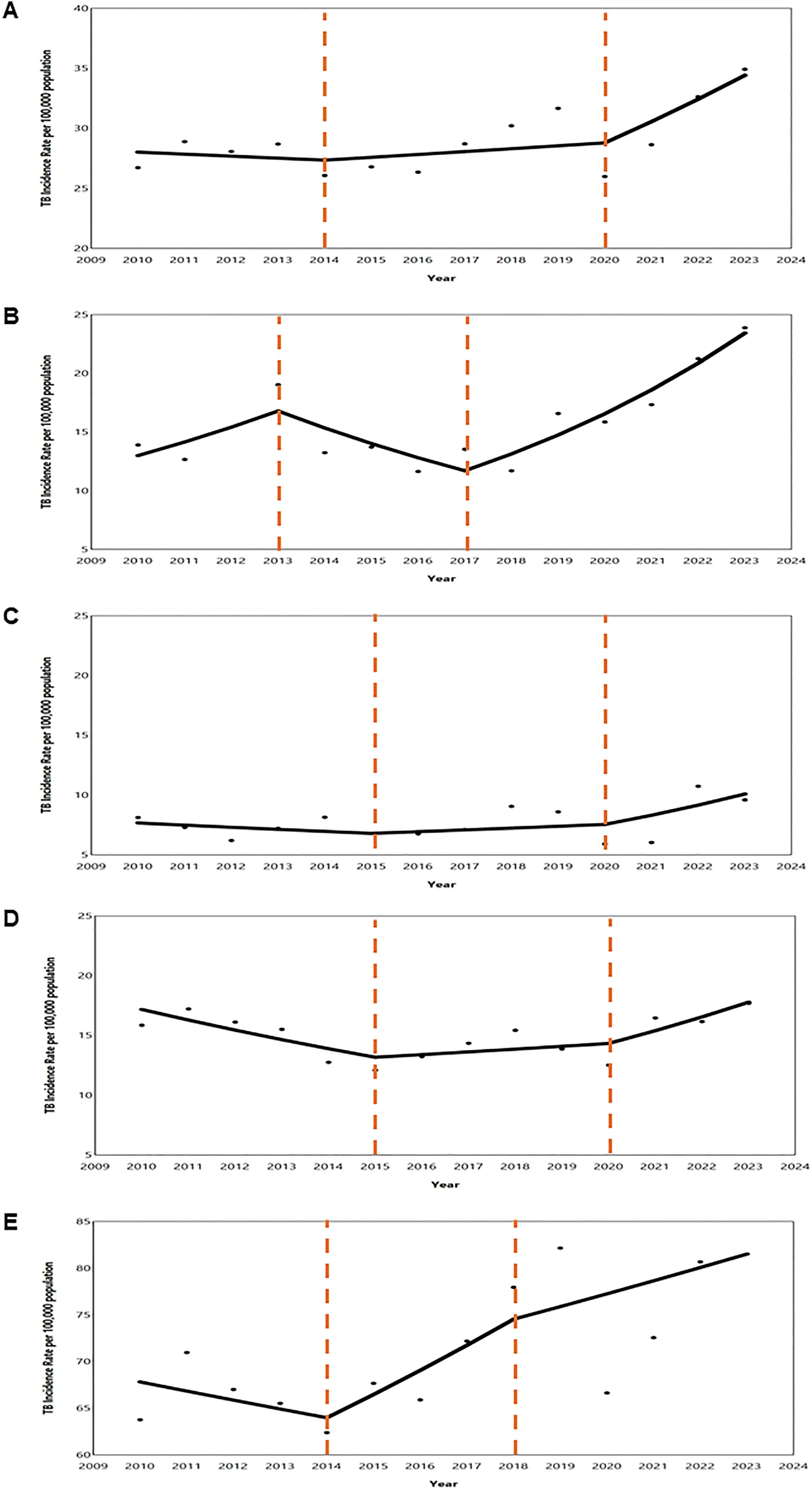
Temporal evolution of the annual incidence rate of tuberculosis among children and adolescente aged 0 to 19 years, in Rio de Janeiro State, Brazil, 2010−2023. Note: (A) overall population (0 to 19 years), (B) 0 to 4 years, (C) 5 to 9 years, (D) 10 to 14 years, (E) 15 to 19 years, TB: tuberculosis. Points represent the observed incidence rates, solid lines represent the Joinpoint regression estimates, and dashed orange vertical lines indicate joinpoints (years with changes in trend were identified).

Between 2010 and 2023, a total of 18,544 tuberculosis cases were notified among children and adolescents in Rio de Janeiro State. Joinpoint analysis of the historical series identified three distinct trend periods: an initial decreasing trend from 2010 to 2014, a stable trend from 2014 to 2020, and a subsequent increasing trend from 2020 to 2023. A similar three-segment pattern was observed in the age-stratified analyses, however, the magnitude and statistical significance of the trends differed across age groups. The most pronounced and statistically significant increase in the final trend segment was observed among children aged 0–4 years, whereas the increases in the older age groups were not statistically significant (Fig 1).

The analysis of Annual Percent Change by age group revealed distinct temporal patterns across the study period. For the overall population aged 0–19 years, a significant increase was observed in the most recent segment (2020–2023), with an APC of 6.13% (95% CI: 1.0–11.42). The 0–4-year age group exhibited pronounced fluctuations, with an initial increasing trend, followed by a decline and a subsequent statistically significant increase in the final segment (APC = 12.3%; 95% CI: 6.6–18.2). In contrast, the 5–9, 10–14, and 15–19-year age groups showed a decreasing trend in the first segment. Although positive APC estimates were observed in the subsequent segments, these trends were classified as stationary because the corresponding confidence intervals included zero. Overall, the age-stratified analyses indicate that the most pronounced and statistically significant increase in tuberculosis incidence occurred among children aged 0–4 years, whereas the older age groups showed stationary trends. The Average Annual Percent Change for the overall population was 1.60% (95% CI: 0.15–3.06; *p* = 0.030), indicating a modest but statistically significant increase over the entire study period. In contrast, the AAPC values for all age-specific groups were not statistically significant, indicating stationary overall trends (Table 1).

**Table 1:** Distribution of annual percent change and average annual percent change in tuberculosis incidence per 100,000 population among children and adolescents aged 0 to 19 years, by age group in Rio de Janeiro State, Brazil, 2010 - 2023.

Age group	APC (95% CI)	AAPC (95% CI)
0 to 19 years		1.60* (0.1 to 3.1)
1º segment (2010–2014)	−0.6 (−3.6 to 2.5)	
2º segment (2014–2020)	0.86 (−1.3 to 3.0)	
3º segment (2020–2023)	6.13* (1.0 to 11.4)	
0 to 4 years		4.64 (−1.1 to 10.6)
1º segment (2010–2013)	8.9 (−7.5 to 28.3)	
2º segment (2013–2017)	−8.6 (−23.3 to 8.8)	
3º segment (2017–2023)	12.3* (6.6 to 18.2)	
5 to 9 years		2.14 (−6.2 to 11.2)
1º segment (2010–2015)	−2.4 (−13.6 to 10.3)	
2º segment (2015–2020)	2.1 (−14.7 to 22.3)	
3º segment (2020–2023)	10.2 (−16.9 to 46.0)	
10 to 14 years		0.27 (−3.7 to 4.4)
1º segment (2010–2015)	−5.2 (−10.5 to 0.4)	
2º segment (2015–2020)	1.7 (−7.1 to 11.3)	
3º segment (2020–2023)	7.5 (−5.7 to 22.4)	
15 to 19 years		1.43 (−3.3 to 6.4)
1º segment (2010–2014)	−1.4 (−10.3 to 8.3)	
2º segment (2014–2018)	3.9 (−10.3 to 20.3)	
3º segment (2018–2023)	1.8 (−4.6 to 8.6)	

APC: annual percent change; AAPC: average annual percent change; (95%CI): 95% confidence interval; *: statistically significant. Source: prepared by the authors.

When stratifying the historical series into the three trend periods identified by the regression model for the age group from 0 to 19 years, significant differences were observed in the distribution of the sociodemographic and clinical variables analyzed (Table 2).

**Table 2:** Sociodemographic and clinical characteristics of reported cases of tuberculosis among children and adolescents aged 0 to 19 years in Rio de Janeiro State, Brazil, stratified by time periods: 2010–2014, 2015–2019, and 2020–2023.

Sociodemographic and clinical characteristics	Total N	Period			P
		2010-2014 N (%)	2015-2019 N (%)	2020-2023 N (%)	
**Sex**					
Female	7903	2952 (43.97)	2759 (41.94)	2192 (41.75)	0.019^*^
Male	10640	3762 (56.03)	3820 (58.06)	3058 (58.25)	
**Race/skin color**					
White	4793	2009 (29.92)	1610 (24.47)	1174 (22.36)	<0.001^*^
Black	4025	1401 (20.87)	1378 (20.95)	1246 (23.73)	
Yellow	150	53 (0.79)	55 (0.84)	42 (0.80)	
Mixed	7701	2499 (37.22)	2842 (43.20)	2360 (44.94)	
Indigenous	43	18 (0.27)	16 (0.24)	9 (0.17)	
Ign/Not filled	1832	734 (10.93)	678 (10.30)	420 (8.00)	
**Beneficiary of a cash transfer program**					
Yes	1369	6 (0.09)	636 (9.67)	727 (13.85)	<0.001^*^
No	7628	177 (2.64)	4171 (63.40)	3280 (62.46)	
Ign/Not filled	9547	6531 (97.27)	1772 (26.93)	1244 (23.69)	
**Clinical Form**					
Pulmonary	15269	5453 (81.22)	5476 (83.23)	4340 (82.65)	0.013^*^
Extrapulmonary	2541	957 (14.25)	875 (13.30)	709 (13.50)	
Pulmonary and extrapulmonary	734	304 (4.53)	228 (3.47)	202 (3.89)	
**HIV**					
Positive	689	320 (4.77)	233 (3.54)	136 (2.59)	<0.001^*^
Negative	13004	3710 (55.26)	4983 (75.74)	4311 (82.10)	
In progress	586	389 (5.79)	102 (1.55)	95 (1.81)	
Not realized	4265	2295 (34.18)	1261 (19.17)	709 (13.50)	
**DOT**					
Yes	7236	2540 (37.83)	2650 (40.28)	2046 (38.96)	<0.001^*^
No	7909	3694 (55.02)	2364 (35.93)	1851 (35.25)	
Ign/Not filled	3399	480 (7.15)	1565 (23.79)	1354 (25.79)	
**Outcome**					
Care	12387	4605 (68.59)	4608 (70.04)	3174 (60.45)	<0.001^*^
Lost to follow-up	2761	964(14.36)	1042 (15.84)	755 (14.38)	
Death from tuberculosis	230	95 (1.42)	62 (0.94)	73 (1.39)	
Death from other causes	170	61 (0.91)	65 (0.99)	44 (0.84)	
Transfer	1205	509 (7.58)	360 (5.47)	336 (6.40)	
RR TB	190	66 (0.99)	78 (1.18)	46 (0.87)	
Change of regimen	24	1 (0.01)	9 (0.14)	14 (0.27)	
Failure	10	2 (0.02)	4 (0.06)	4 (0.08)	
Other	1567	411(6.12)	351 (5.34)	805 (15.33)	

Ign: ignored; DOT: directly observed treatment; RR TB: rifampicin resistance; HIV: human immunodeficiency vírus; ^*^ statistically significant. Source: prepared by the authors.

Note: Percentages represent the proportion of cases within each time interval (2010–2014, 2015–2019, and 2020–2023). Values are column-standardized and therefore sum to 100% within each period.

The distribution of tuberculosis cases by sex differed significantly across the study periods (p = 0.019), with males consistently accounting for the majority of reported cases. The proportion of cases among individuals identified as white decreased over time, while those identified as black and mixed race showed a progressive increase. This shift in racial/ethnic distribution was statistically significant (p < 0.001). The proportion of patients receiving government benefits rose from 0.09% to 13.8% (p < 0.001). Pulmonary forms remained predominant (p = 0.013), and HIV testing coverage improved, with a reduction in untested cases (p < 0.001). The use of directly observed treatment increased initially but decreased in the final period (p < 0.001). Treatment outcomes also changed, with cures increasing initially and then decreasing in the final period (p < 0.001).

Similarly, significant differences in the MHDI, infant mortality rate, and Gini index were observed across the three trend periods (2010–2014, 2015–2019, and 2020–2023) defined according to the Joinpoint analysis of the overall population aged 0–19 years (Table 3).

**Table 3:** Human development and social inequality indicators in Rio de Janeiro State according to Joinpoint-defined tuberculosis trend periods among children and adolescents (2010–2023).

Human Development Indices	Period			p
	2010-2014	2015-2019	2020-2023	
	Mean (± SD)	Mean (± SD)	Mean (± SD)	
MHDI	0.7705 (±0.009)	0.7958 (±0.010)	0.7775 (±0.010)	0.007 ^*^
Per capita income	935.76 (±62.93)	946.18 (±67.89)	935.37 (±24.89)	0.947
Infant mortality	12.97 (±0.75)	11.19 (±0.59)	12.99 (±0.89)	0.004 ^*^
Gini index	0.536 (±0.031)	0.528 (±0.019)	0.546 (±0.013)	0.035 ^*^

MHDI: municipal human development index; SD: standard deviation ^*^ statistically significant. Source: prepared by the authors.

The MHDI increased from 0.7705 (±0.009) in the first period to 0.7958 (±0.010) in the second, followed by a decline to 0.7775 (±0.010) in the third, with a statistically significant variation (p = 0.007). Infant mortality showed a temporary improvement, decreasing to 11.19 (±0.59) in the second period, but returned to initial levels in the third (12.99 ± 0.89), also with significant variation across periods (p = 0.004). The Gini index, decreased slightly between the first and second periods (0.536 ± 0.031 to 0.528 ± 0.019), but increased again in the third (0.546 ± 0.013), indicating a rise in socioeconomic disparity (p = 0.035). In contrast, per capita income remained stable throughout the study period, with no statistically significant change (p = 0.947).

## Discussion

This study emerged from the need for periodic evaluations of tuberculosis incidence rates in order to provide health authorities with reliable data to guide the development of more effective strategies for disease control, particularly among vulnerable populations such as children and adolescents. The findings indicate that the temporal trend in tuberculosis incidence varied throughout the study period, with distinct patterns observed across different age groups. For the overall population aged 0–19 years, Joinpoint analysis identified a non-significant decreasing trend between 2010 and 2014, followed by a stationary trend from 2015 to 2019 and a significant increasing trend from 2020 to 2023. Among the age-specific groups, the most pronounced increase was observed in children aged 0–4 years, who exhibited a significant increase in the final segment (APC = 12.3%), indicating a reversal of the previous declining trend. These results highlight the importance of age-specific surveillance and the need for targeted public health interventions to curb the increasing burden of tuberculosis in the pediatric population.

The recent resurgence coincided with changes in the clinical and epidemiological profile, including a higher proportion of cases among Black and mixed-race children, a decline in the cure rate, and reduced use of Directly Observed Treatment, suggesting challenges in tuberculosis control and continuity of care. Variations in socioeconomic indicators, including the MHDI, infant mortality rate, and Gini index, were also observed during this period. Although these changes were relatively small and should be interpreted as contextual rather than causal, they occurred in parallel with the resurgence of tuberculosis and may reflect broader social and health system challenges.

The trend periods identified in this study coincide with important sociopolitical and public policy changes that, although not implying a causal relationship, provide a relevant contextual framework for interpreting the observed epidemiological patterns. During the first segment (2010–2014), a non-significant reduction in tuberculosis incidence among children and adolescents in Rio de Janeiro State was observed. This period overlapped with the expansion of social protection policies, particularly the Bolsa Família Program [12], increased primary health care coverage through the Family Health Strategy [13–15], and the consolidation of the decentralization of the National Tuberculosis Control Program. During this process, Family Health Strategy teams progressively incorporated tuberculosis surveillance, active case finding, treatment follow-up, and contact investigation into routine primary care activities, strengthening the integration between primary health care and tuberculosis control [16].

This interpretation is supported by a cohort study conducted in Rio de Janeiro State between 2004 and 2013, which found that greater access to health services, particularly through the Family Health Strategy, was associated with improved tuberculosis treatment success rates [13]. The same study also reported that broader primary healthcare coverage was associated with lower tuberculosis incidence, highlighting the potential contribution of primary care to tuberculosis prevention and control [13,17].

In the second trend segment (2015–2019), tuberculosis incidence among children and adolescents remained statistically stationary. During the same period, the socioeconomic indicators evaluated in this study showed modest improvements, including an increase in the Municipal Human Development Index (MHDI) and favorable changes in the infant mortality rate and Gini index. Similarly, national studies have reported overall stability in tuberculosis incidence between 2015 and 2019, despite regional heterogeneity and localized increases in cases in socially vulnerable territories after 2016 [4,10,18–21].

In 2020, this context was further marked by the onset of the COVID-19 pandemic, one of the most severe global health crises in recent history. During this period, Brazil experienced worsening socioeconomic conditions, disruptions in health services, and reduced access to social protection [22–24]. These circumstances disproportionately affected vulnerable populations, particularly children and adolescents, and provide important context for interpreting the epidemiological changes observed after 2020.

During the most recent study period (2020–2023), a statistically significant increase in tuberculosis incidence among children and adolescents was observed, particularly among those under five years of age. This period coincided with the aftermath of the COVID-19 pandemic, when persistent disruptions in health services, reduced BCG vaccination coverage, and worsening social vulnerability were reported in Brazil [18,22]. Although the national COVID-19 vaccination campaign represented an important public health achievement, primary healthcare services continued to face substantial operational challenges during this period [22–24]. Previous studies have reported that these disruptions were associated with delays in tuberculosis diagnosis and reduced access to tuberculosis control activities among children and adolescents, providing important context for interpreting the increase in incidence observed in the present study [25].

Additionally, socioeconomic indicators such as poverty, income distribution, and social vulnerability have been affected by economic and social changes in Brazil, with potential implications for population health conditions [22]. In this study, variations in the Municipal Human Development Index (MHDI), infant mortality rate, and Gini index were observed across the trend periods identified by the Joinpoint model. However, these indicators should be interpreted as contextual measures rather than direct determinants of the observed tuberculosis trends. Although MHDI remained within the high human development category throughout the study period, aggregate socioeconomic indicators may not fully capture persistent social inequalities and vulnerabilities affecting specific population groups. Therefore, these findings should be interpreted within the broader social and health system context, recognizing that tuberculosis epidemiology is shaped by multiple interacting determinants, including social inequalities, conditions of social vulnerability, and the organization of tuberculosis prevention, diagnosis, and control services.

The sharper increase in tuberculosis incidence among children under five is particularly concerning, as this age group is more susceptible to progression from infection to active disease and therefore serves as an important indicator of recent tuberculosis transmission. This finding should be interpreted in the context of reported disruptions in BCG vaccination, contact investigation, and access to health services during the study period. Previous studies have emphasized that maintaining high BCG vaccination coverage, ensuring timely diagnosis, and expanding tuberculosis preventive treatment are essential strategies for reducing the burden of childhood tuberculosis [26].

Changes in the sociodemographic distribution of tuberculosis cases deserve special attention. An increase in the proportion of cases reported among individuals identified as Black or mixed-race was observed over the study period, indicating a growing representation of these population groups among notified tuberculosis cases. These findings reinforce the social dimension of tuberculosis and are consistent with ecological studies reporting higher tuberculosis incidence in areas characterized by greater social vulnerability and social inequalities [19]. Additionally, a higher proportion of individuals were reported as beneficiaries of government cash transfer programs in the later study period. However, this finding should be interpreted with caution, as this variable was introduced following revisions to the SINAN notification form in 2014/2015, which may have affected its completeness and comparability across the study periods.

The reduction in the coverage of Directly Observed Treatment (DOT) and the cure rate observed in the most recent period may indicate challenges in tuberculosis case follow-up and continuity of care within primary healthcare services. These findings are consistent with reports describing disruptions in health service delivery during periods of health and social crises, which may have affected the implementation of tuberculosis control activities [10]. In addition, tuberculosis mortality among children and adolescents, after declining during the earlier study periods, increased in the most recent period, consistent with national trends reported in Brazil [5,19]. Together, these findings highlight the need to strengthen tuberculosis control strategies, ensure continuity of care, and maintain systematic monitoring of key programmatic indicators to improve outcomes among children and adolescents.

Since December 2013, national guidelines have recommended routine HIV testing for all individuals diagnosed with tuberculosis. Accordingly, an increase in HIV testing among children and adolescents was expected and partially confirmed in this study. However, universal testing remains inconsistent, falling short of the policy’s goals. Ensuring routine HIV screening in coinfected individuals is critical, given its role in reducing mortality and morbidity associated with TB-HIV coinfection [27].

Although the findings of this study do not support a direct causal relationship between the political and economic context and the observed changes in tuberculosis incidence, the temporal overlap between these events provides an important contextual framework for interpreting the epidemiological trends. The 2015–2016 economic recession, reductions in social and health investments, and disruptions to health services during the COVID-19 pandemic coincided with changes in the epidemiological profile of tuberculosis in Rio de Janeiro State. Previous studies have suggested that these events were associated with challenges in maintaining tuberculosis control activities, particularly those related to primary healthcare, epidemiological surveillance, and access to health services [10,28].

This study has some limitations that should be considered when interpreting the findings. First, the use of secondary data from health information systems may have introduced biases related to underreporting, incomplete records, reporting delays, and inconsistencies in data quality. In addition, sensitivity analyses for missing data were not performed. Second, as an ecological time-series study, the analyses identify temporal trends and statistical associations but do not allow causal inference at the individual level. Therefore, the statistical significance of the Joinpoint analyses should be interpreted together with the magnitude and direction of the Annual Percent Change (APC) and Average Annual Percent Change (AAPC), rather than based solely on p-values. Additionally, temporal comparisons of some variables should be interpreted with caution due to changes in the SINAN notification form implemented in 2014/2015, which affected the availability, definition, and completeness of certain variables. Consequently, observed differences across study periods may partially reflect changes in data collection and reporting practices rather than solely underlying epidemiological changes. Finally, official annual MHDI values were unavailable for 2011, 2022, and 2023. Therefore, these values were replaced using mean imputation based on the available annual observations. Although this approach allowed the inclusion of the indicator throughout the study period, it may have reduced temporal variability and masked short-term socioeconomic changes.

Despite these limitations, this study has important strengths. By analyzing a 14-year time series of pediatric tuberculosis in Rio de Janeiro State, it provides a comprehensive assessment of changes in the epidemiological profile of the disease. The findings highlight an increase in tuberculosis incidence after 2020, particularly among children under five years of age, identify weaknesses in the health system response, as reflected by declining cure rates and reduced use of directly observed treatment, and underscore the influence of social vulnerability, evidenced by the growing proportion of cases among Black and mixed-race children. Together, these findings provide relevant evidence to inform public health policies and reinforce the need to strengthen tuberculosis control strategies, ensure continuity of care, and address the social determinants of health to reduce the burden of childhood tuberculosis.

## Conclusions

The findings of this study indicate that tuberculosis incidence among children and adolescents in Rio de Janeiro State varied between 2010 and 2023, with a statistically significant increase observed from 2020 to 2023, particularly among children under five years of age. During the same period, changes were observed in epidemiological, programmatic, and socioeconomic indicators, including Directly Observed Treatment coverage, cure rates, the Municipal Human Development Index, infant mortality rate, and the Gini index, highlighting the complexity of the context in which these trends occurred.

Taken together, these findings reinforce the importance of continuous epidemiological surveillance of childhood tuberculosis and the monitoring of key programmatic indicators to support timely public health responses. Although causal relationships cannot be established, the observed patterns underscore the need to strengthen tuberculosis prevention, early diagnosis, continuity of care, and intersectoral actions addressing social vulnerability, particularly among children under five years of age and other high-risk populations.

## Supporting information

S1 File Tables generated by the Tabnet system on May 8, 2024. (XLSX)
